# Adult Onset Langerhans Cell Histiocytosis: Clinical Characteristics and Treatment Outcomes

**DOI:** 10.1007/s44228-023-00034-w

**Published:** 2023-02-24

**Authors:** Eren Arslan Davulcu, Nur Soyer, Zühal Demirci, Ajda Güneş, Filiz Vural, Fahri Şahin, Mahmut Töbü, Serra Kamer, Mine Hekimgil, Güray Saydam

**Affiliations:** 1 University of Health Sciences Bakırkoy Dr. Sadi Konuk Training and Research Hospital, Hematology Clinic, Zuhuratbaba Mah, Dr. Tevfik Sağlam Cad No:11, Bakırköy, 34147 Istanbul, Turkey; 2 Faculty of Medicine, Hematology Department, Ege University, İzmir, Turkey; 3 Faculty of Medicine, Radiation Oncology Department, Ege University, İzmir, Turkey; 4 Faculty of Medicine, Pathology Department, Ege University, İzmir, Turkey

**Keywords:** Histiocytic disorders, Langerhans cell histiocytosis, Cladribine

## Abstract

**Purpose:**

Langerhans cell histiocytosis (LCH) is a rare disease that can affect all tissues and organs. Our study evaluated the clinical characteristics and treatment outcomes of adult-onset LCH patients in a tertiary center.

**Materials and Methods:**

Adult patients diagnosed with LCH were retrospectively evaluated. Their initial symptoms, stratification according to disease involvement, treatment details, treatment responses, and overall and progression-free survival (PFS) were analyzed.

**Results:**

Thirty-three patients were included. There were 21 single system LCH, 10 multisystem LCH, and 2 pulmonary LCH patients. Patients with single system unifocal involvement were successfully treated with local therapies such as surgery and radiotherapy. Most of the multisystem LCH patients and patients with single system multifocal involvement were treated with systemic chemotherapy. Cladribine was the first choice in 10 out of 11 patients who received chemotherapy. Among all patients, the overall response rate (ORR) was 97%. Among those who had cladribine in the first-line the ORR was 81%. All these patients achieved a complete remission and were alive at the last visit. The median follow-up was 38 (range, 2–183) months. The median PFS has not yet been reached. Ten-year PFS was 90.9%.

**Conclusion:**

Besides successful local treatments with surgery and radiotherapy, our study provides information for front-line cladribine treatment.

## Introduction

Langerhans cell histiocytosis (LCH) is a rare systemic disease that is characterized by the accumulation of CD1a/Langerin-positive cells in various tissues and organs. Recent developments have shown that the histiocytes in LCH originate from myeloid dendritic cells [1]. Furthermore, the presence of activating, somatic *BRAF* mutations in most cases indicates that it is a clonal disease [2]. The annual incidence of LCH ranges from 2.6 to 8.9 cases per million children younger than 15 years [3–6] and, in adults, 0.07 cases per million per year for disseminated disease [7]. Any organ or system can be affected by LCH, and its location determines the clinical presentation and severity of the disease. Bones, skin, lungs and pituitary gland involvement are common. Treatment strategies range from observation without any treatment to systemic intensive chemotherapy and radiotherapy [8].

This study reports the clinical features, treatment details, and outcomes of adult LCH patients diagnosed in our center.

## Materials and Methods

### Patients

Thirty-three adult patients who were diagnosed with, treated, and followed up for LCH in our center between 2000 and 2020 were retrospectively evaluated. This study was approved by the local ethics committee and was conducted according to the principles of the Declaration of Helsinki. All patients provided written informed consent.

### Diagnosis and Clinical Evaluation

Patients were diagnosed with LCH according to specific histopathological findings and immunohistochemical positivity for at least two of the markers S100, CD1a and Langerin (CD207). All patients were screened by total bone X-ray survey and/or bone scintigraphy, abdominal and chest computerized tomography, cranial magnetic resonance imaging and, some of the patients, by positron emission tomography. Bone marrow aspiration and biopsy were performed in all patients in order to evaluate the possible bone marrow infiltration. Complete blood count, blood chemistry (including total protein, albumin, bilirubin, alanine aminotransferase, aspartate aminotransferase, alkaline phosphatase, gamma-glutamyl transpeptidase, electrolytes, urea, creatinine, c-reactive protein), erythrocyte sedimentation rate, coagulation studies (prothrombin and activated partial thromboplastin time), thyroid-stimulating hormone and free T4, other pituitary gland hormones if the pituitary gland was involved, and urine strip test were analyzed. Patients were stratified according to the involvement of the disease [9]:

A) Single system LCH (SS-LCH): One organ or system involved. It may be uni- or multifocal
B) Multisystem LCH (MS-LCH): Two or more organs/systems involved
C) Pulmonary LCH: Isolated lung disease
D) Central nervous system (CNS) LCH: Tumorous lesions, neurodegenerative disease

### Treatment

Treatment options were surgery, radiotherapy, and chemotherapy. The initial chemotherapy protocol for our center was mainly subcutaneous cladribine at a dose of 5 mg/m^2^ over five consecutive days, every four weeks for six courses. Only one patient received vinblastine plus prednisolone for the starting course.

Patients were followed-up monthly during the treatment period, every 3 months after the end of the treatment for 1 year, and every 6 months for the following 3 years. After that, patients were only examined in the case of presenting LCH-related symptoms.

### Response

Complete remission (CR) was defined as the disappearance of all LCH-related lesions, partial remission (PR) as a minimum 50% reduction in lesion mass, stable disease (SD) as not meeting other CR and PR criteria, progressive disease (PD) as growth of existing lesions or appearance of new lesions despite treatment. Relapse was defined as development of new lesions or progression of old lesions after response to treatment.

Outcome measures were overall survival (OS), progression-free survival (PFS), treatment-related mortality (TRM), long-term sequelae (such as endocrinologic problems, pulmonary fibrosis, gait disturbance) and secondary malignancies.

### Statistical Analysis

Data were reported as frequency (percentage) or median for categorical and continuous variables. Survival analyses were performed using the Kaplan–Meier method. PFS and OS were calculated from the start of therapy until disease progression or death, or until the last follow-up. IBM SPSS Statistics 25 for Windows was used for statistical analyses.

## Results

A total of 33 adult-onset LCH patients were evaluated. Clinical characteristics are summarized in Table 1. All patients were adult onset, there were no patients with relapsed infancy onset LCH.

**Table 1:** Clinical characteristics

Age at diagnosis, years, median (range)	38 (18–69)
Gender	
Male, number (%) Female, number (%)	24 (72.7%) 9 (27.3%)
Initial symptoms, number (%)	
Swelling Pain (head /extremity/chest pain) Cough Weakness in an extremity Gait and speech impairment Amnesia	9 (27.3%) 7/11/1 (23%/33%/3%) 1 (3%) 1 (3%) 2 (6%) 1 (3%)
Biopsy site, number (%)	
Bone Lung Skin Thyroid Ear Gum Cervix and clitoris Intraabdominal	21 (63.6%) 3 (9%) 2 (6%) 2 (6%) 2 (6%) 1 (3%) 1 (3%) 1 (3%)
Disease classification, number (%)	
Single system LCH Multisystem LCH Pulmonary LCH Central nervous system LCH	21 (63.6%) 10 (30.3%) 2 (6.6%) 0 (0%)
Organ involvement, number (%)	
Bone Unifocal Multifocal Lung Skin Central nervous system Thyroid Ear Gum Cervix and clitoris Intraabdominal	23 (69.6%) 14 9 7 (30.4%) 3 (9%) 3 (9%) 2 (6%) 2 (6%) 2 (6%) 1 (3%) 1 (3%)

Out of 3 patients with CNS involvement, 1 had isolated pons, 1 had isolated cerebellum lesion, and 1 had concomitant cerebellum lesion with involvement in the brain parenchyma.

Of the 7 patients with pulmonary lesions, 2 were pulmonary LCH and the other 5 were MS-LCH.

All patients with single-system unifocal involvement were treated with local therapies, such as surgery and radiotherapy. Three patients with multisystem involvement were treated with local therapies too. Treatment details are presented in Table 2.

**Table 2:** Treatment details

Only surgery	6 (18.1%)
Surgery + radiotherapy	14 (42.4%)
Only steroid	1 (3%)
Only radiotherapy	1 (3%)
Cladribine	7 (21.2%)
Cladribine + radiotherapy	2 (6%)
Vinblastine/prednisolone + cladribine	1 (3%)
Cladribine + radiotherapy + lenalidomide + Vinblastine/prednisolone	1 (3%)

Four patients with single-system involvement of LCH received systemic therapy because of its multifocal distribution (Table 3).

**Table 3:** Treatment choices according to disease status (numbers)

	Single system unifocal	Single system multifocal	Multisystem	Pulmonary
Systemic chemotherapy	0	4	7	0
Local treatment (Including surgery and radiotherapy)	15	2	3	1
Steroid	0	0	0	1

Cladribine was the first choice in 10 out of 11 patients who received chemotherapy. The median number of cycles of cladribine administered was 6 (range, 1–6). One patient received vinblastine + steroid as initial therapy because there was a temporary disruption in the supply of cladribine. Since this patient did not respond to vinblastine + steroid treatment, cladribine was administered in the second-line and a CR was achieved at the end of 6 cycles.

The overall response rate (ORR) was 97% among all patients. Thirty-one patients (94%) achieved CR, 1 (3%) remained in SD, and 1 (3%) showed a PD. ORR was 81% among patients who received cladribine in the first-line treatment, and the treatment response of all of these patients were CR.

The relapse rate was low and occurred three times in only 1 patient. That patient had MS-LCH involving the left mandible, pons, lung, and left tibia. Her LCH first occurred in the mandible and was treated with surgical excision followed by radiotherapy. For the first and second relapses, she received radiotherapy again for the mandible and pons. In the third relapse, the lung was also affected and therefore she received vinblastine + steroid chemotherapy. She has been in remission for five years.

The most common treatment-related adverse event was febrile neutropenia, which was seen in 10 (30.3%) patients (grade 1 in 4, grade 2 in 3, grade 3 in 3). Also, one patient had vinblastine-induced grade-2 neurotoxicity. Three patients developed diabetes insipidus (9%) in the long term. Two of them remain on desmopressin-replacement therapy.

Two patients had a history of malignancy. One of them was diagnosed with both LCH and thyroid cancer concomitantly from the same thyroid biopsy, and was treated with radioactive iodine after surgery. Because LCH was limited to the thyroid, treatment was limited to surgery. The other patient had a LCH lesion limited to the thyroid gland and was treated only by surgical excision. The same patient was diagnosed with lung cancer 3 years after being diagnosed with LCH. He is still receiving systemic chemotherapy. There is no evidence for recurrence of LCH.

**Fig. 1:**
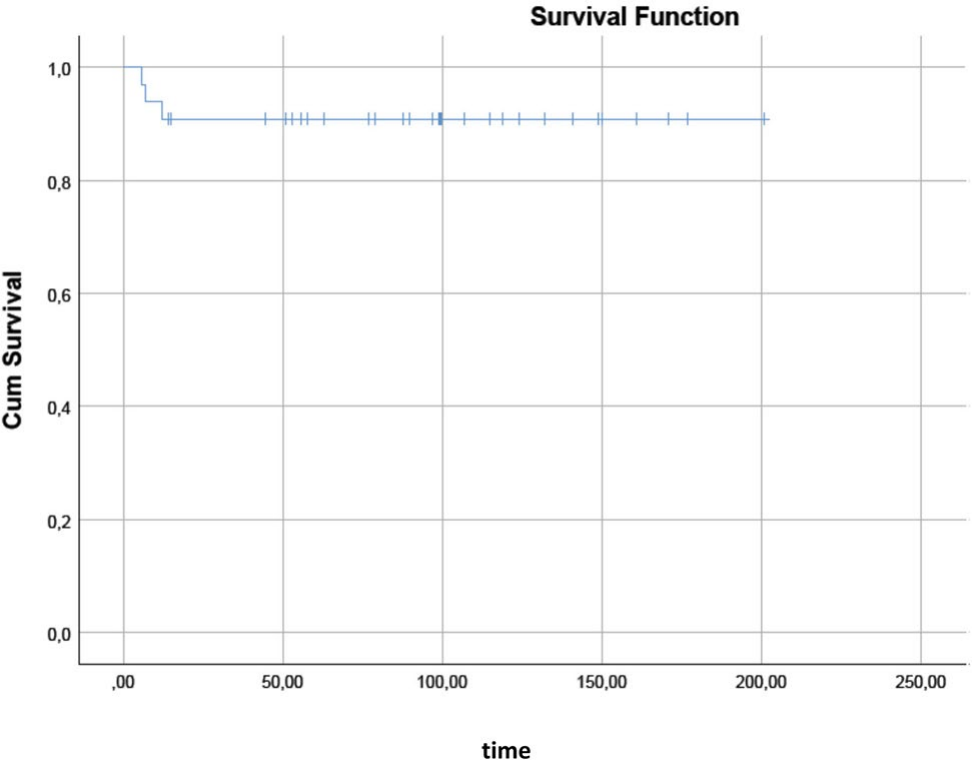
PFS (progression-free survival) curve of all patients (months)

All of our patients were alive at the time of the last visit. The OS of our study population was 100% with a median follow-up of 38 months (2–183 months). The median PFS has not yet been reached, and the 10-year PFS was 90.9% (Fig. 1). There was no treatment-related mortality.

## Discussion

LCH is a rare disease in the whole population and is even rarer in adults compared to the pediatric age group. The available literature is generally based on data from pediatric patients. Therefore, studies on adult LCH disease are important.

LCH can present a wide range of symptoms. The most prominent complaints in our patient group were pain and swelling. In cases with such unexplained symptoms which cannot be diagnosed by classical methods, rare diseases such as LCH should also be considered, and biopsy should not be delayed. Bone was the most frequently involved and the most biopsied tissue in our patient group.

Thyroid gland LCH is a type of involvement mentioned in the literature, and there were two such cases in our patient group. One of these patients had a single-system disease and was treated with a thyroidectomy. The other had multi-system disease with involvement of thyroid and gums. This case is interesting because the patient had both papillary thyroid carcinoma (PTC) and LCH in the same thyroid biopsy. Following the thyroidectomy, the patient was treated with radioactive iodine and is still in remission for both diseases. Co-existence or sequential presentation of LCH and PTC has been reported before [10, 11]. BRAF gene mutation can be detected in one or both of these tumors.

LCH limited to the female genital tract is also an extremely rare presentation [12–15]. Our case can be classified as 'pure' LCH of the female genital tract without any other spreading. She was treated with surgical excision of the lesions in the cervix and the clitoris, and then received radiotherapy for involved sites. She has not shown any signs of local or systemic recurrence so far.

LCH involvement in the abdomen is also rare, except for the liver and spleen. One of our patients who had diffuse abdominal masses of up to 13 cm was diagnosed with LCH via an intraabdominal biopsy. New masses developed on her neck after two courses of cladribine treatment, when the course was interrupted for the performance of a mass biopsy.

There was a single system LCH predominance (63.3%) in our study group which is unlike the other adult studies [16, 17], but similar to one pediatric study [18]. More than half of our patients (69.6%) had bone lesions, which is quite similar to the other adult LCH study (62%) [16, 17].

The number of existing studies examining the efficacy of cladribine in the first-line treatment of adult LCH is inadequate. Our study is also important in terms of filling in this gap. A recent comprehensive study evaluated the effectiveness of front-line cladribine in 29 and subsequent lines in 9 adult patients. In that patient group, the ORR of cladribine in the front-line setting was 83% [19], which is similar to our result (81%). In a single-center study consisting of 7 patients, durable CR were maintained in six patients after cladribine treatment (86%) [20].

Although there are differences in the choice of first-line chemotherapy and patient characteristics, our OS and PFS rates were similar to those of a previous adult LCH study [16]. In another cladribine report for adult LCH patients, 5-year OS and PFS were 75% and 58%, respectively [19]. The reason for the relatively low survival rates may be that there were more multisystem-LCH patients in that patient group compared to ours (82 versus 30.3%).

In the study of Cao et al. with 266 newly diagnosed adult LCH patients, cytarabine-based therapy predicted favorable OS [21]. As supported by Goyal et al. [22] and Doberauer et al. [17], cladribine- and cytarabine-based chemotherapies produced the best results. Although our center has no experience with the use of cytarabine in LCH, these results are instructive.

BRAF-V600E mutation testing results were not included in our study because the test could be performed only on a very limited number of cases.

## Conclusion

Since LCH is a rare disease, we think it is important to share real-life data about the disease. The correct staging of the disease at the time of diagnosis determines the treatment approach. As shown in our study, the success of both local and systemic treatments is satisfactory, especially with cladribine.

## Data Availability

Raw data were generated at Ege University Mecical Faculty. Derived data supporting the findings of this study are available from the corresponding author [EAD] on request.
